# Editorial

**DOI:** 10.3897/zookeys.540.6506

**Published:** 2015-11-26

**Authors:** Marc De Meyer, Anthony R. Clarke, M. Teresa Vera, Jorge Hendrichs

**Affiliations:** 1 Royal Museum for Central Africa, Tervuren, Belgium; 2 Queensland University of Technology, Brisbane, Australia; 3 Cátedra Terapéutica Vegetal, Facultad de Agronomía y Zootecnia (FAZ), Universidad National de Tucumán (UNT), San Miguel de Tucumán, Argentina; 4 Consejo Nacional de Investigaciones Científicas y Técnicas (CONICET), Argentina; 5 Joint FAO/IAEA Division of Nuclear Techniques in Food and Agriculture, Vienna International, Centre, Vienna, Austria

Tephritid fruit flies (Diptera: Tephritidae) are considered by far the most important group of horticultural pests worldwide. Female fruit flies lay eggs directly into ripening fruit, where the maggots feed causing fruit loss. Each and every continent is plagued by a number of fruit fly pests, both indigenous as well as invasive ones, causing tremendous economic losses. In addition to the direct losses through damage, they can negatively impact commodity trade through restrictions to market access. The quarantine and regulatory controls put in place to manage them are expensive, while the on-farm control costs and loss of crop affect the general well-being of growers. These constraints can have huge implications on loss in revenues and limitations to developing fruit and vegetable-based agroindustries in developing, emergent and developed nations.

Because fruit flies are a global problem, the study of their biology and management requires significant international attention to overcome the hurdles they pose. The Joint Food and Agriculture Organisation / International Atomic Energy Agency (FAO/IAEA) Programme on Nuclear Techniques in Food and Agriculture has been on the foreground in assisting Member States in developing and validating environment-friendly fruit fly suppression systems to support viable fresh fruit and vegetable production and export industries. Such international attention has resulted in the successful development and validation of a Sterile Insect Technique (SIT) package for the Mediterranean fruit fly.

Although demands for R&D support with respect to Mediterranean fruit fly are diminishing due to successful integration of this package into sustainable control programmes against this pest in many countries, there were increasing demands from Member States in Africa, Asia and Latin America, to address other major fruit fly pests and a related, but sometimes neglected issue of tephritid species complexes of economic importance. Any research, whether it is basic or applied, requires a taxonomic framework that provides reliable and universally recognized entities and names. Among the currently recognized major fruit fly pests, there are groups of species whose morphology is very similar or identical, but biologically they are distinct species. As such, some insect populations that are grouped taxonomically within the same pest species, display different biological and genetic traits and show reproductive isolation which suggest that they are different species. On the other hand, different species may have been taxonomically described, but there may be doubt as to whether they actually represent distinct biological species or merely geographical variants of the same species. This uncertain taxonomic status has practical implications on the effective development and use of the SIT against such complexes, particularly at the time of determining which species to mass-rear, and significantly affects international movement of fruit and vegetables through the establishment of trade barriers to important agricultural commodities which are hosts to these pest tephritid species.

A Consultants’ Meeting, organized by the IAEA and held on 6-10 July 2009 in Vienna, Austria, discussed a number of major fruit fly complexes and prioritised them as to their economic importance, regional importance and potential for SIT application. Three complexes and a suspected complex were identified to be of significant importance that needed to be resolved to facilitate world agricultural trade and SIT programmes. They were:

- Anastrepha fraterculus
- *Bactrocera cucurbitae* (suspected complex)
- Bactrocera dorsalis
- *Ceratitis* FAR complex (*Ceratitis fasciventris*, *Ceratitis anonae*, *Ceratitis rosa*)

Based on this outcome, a FAO/IAEA Co-ordinated Research Project (CRP) on “Resolution of Cryptic Species Complexes of Tephritid Pests to Overcome Constraints to SIT Application and International Trade” was initiated and officially launched at the first Research Coordination Meeting (RCM) in Vienna on 2–6 August 2010. When addressing the status for each of these complexes, an integrative taxonomic approach was followed, whereby researchers used multiple, independent lines of evidence to delimit the species boundaries. These independent lines included traditional morphology, morphometrics and geomorphometics, developmental physiology, pre- and postzygotic mating incompatibility, karyology, chemoecology, and a wide range of molecular techniques such as multi-locus markers and microsatellites among others. Over a six year period, researchers from more than 20 countries looked at a wide range of different aspects of species delimitation for the priority complexes and presented their findings and research progress during consecutive RCMs in Brisbane, Australia (February 2012), Tucumán, Argentina (August 2013) and La Réunion, France (June 2015). This volume presents the result of this collaborative and integrated approach to resolve the species complexes and clearly demonstrates the advantage of combining efforts, expertise and team-working when addressing such a complex issue as species boundaries. In total, 25 articles are included in this issue. Each paper was peer-reviewed by at least one, but usually two or more independent experts. We would like to thank the many reviewers for their valuable input and assistance in improving the contents of many of the papers. A synthesis of the findings is given in the Introductory Summary Paper, which also includes references to the many papers published elsewhere by CRP participants during the life of the project.

Regrettably, during the period that this CRP was running, we lost two prominent and leading scientists who were involved in fruit fly research for a long time and contributed to species recognition: Serge Quilici and Peter Teal.

Serge Quilici (1955–2015) was a senior researcher at CIRAD (Centre de Coopération International en Recherche Agronomique pour le Développement) based in La Réunion, France. He was well known in the world of fruit fly research, in particular for his involvement in research on fruit fly behaviour, invasive species, interspecific competition, host selection and the role of semiochemicals among others. As a longstanding member of the fruit fly community he coordinated several international projects, supervised many students (in particular from Africa), and kept a number of official functions in international bodies and organisations.

Peter Teal (1953-2015) was a research leader of the chemistry research unit at the Center for Medical, Agricultural and Veterinary Entomology (CMAVE) at USDA-ARS Gainesville, Florida (USA) and Acting Station Director at the Subtropical Horticulture Research Station in Miami. He was mainly known for his outstanding work in the field of isolation and identification of naturally produced compounds that can affect the behaviour and reproduction of insects, including fruit flies. His research on insect physiology was of great importance in the development of control and monitoring strategies for pest species and Peter received international recognition for his research outputs and leadership.

Both researchers were also very warm and kind persons willing to assist and guide fellow scientists in their research. Their guidance but also their friendliness and companionship will be sorely missed by all of us. We, therefore, dedicate this issue to the memory of these two scientists who have shown the path to several of us and upon whose work we have continued to explore the boundaries of these species complexes.

The Editors

September 2015

